# Resting State BOLD Functional Connectivity at 3T: Spin Echo versus Gradient Echo EPI

**DOI:** 10.1371/journal.pone.0120398

**Published:** 2015-03-06

**Authors:** Piero Chiacchiaretta, Antonio Ferretti

**Affiliations:** 1 Department of Neuroscience, Imaging and Clinical Sciences, University “G. d’Annunzio” of Chieti, Chieti, Italy; 2 Institute for Advanced Biomedical Technologies (ITAB), University “G. d’Annunzio” of Chieti, Chieti, Italy; 3 Bioengineering Unit, IRCCS Neuromed, Pozzilli (IS), Italy; University Of Cambridge, UNITED KINGDOM

## Abstract

Previous evidence showed that, due to refocusing of static dephasing effects around large vessels, spin-echo (SE) BOLD signals offer an increased linearity and promptness with respect to gradient-echo (GE) acquisition, even at low field. These characteristics suggest that, despite the reduced sensitivity, SE fMRI might also provide a potential benefit when investigating spontaneous fluctuations of brain activity. However, there are no reports on the application of spin-echo fMRI for connectivity studies at low field. In this study we compared resting state functional connectivity as measured with GE and SE EPI sequences at 3T. Main results showed that, within subject, the GE sensitivity is overall larger with respect to that of SE, but to a less extent than previously reported for activation studies. Noteworthy, the reduced sensitivity of SE was counterbalanced by a reduced inter-subject variability, resulting in comparable group statistical connectivity maps for the two sequences. Furthermore, the SE method performed better in the ventral portion of the default mode network, a region affected by signal dropout in standard GE acquisition. Future studies should clarify if these features of the SE BOLD signal can be beneficial to distinguish subtle variations of functional connectivity across different populations and/or treatments when vascular confounds or regions affected by signal dropout can be a critical issue.

## Introduction

Since its discovery [1–3], functional magnetic resonance imaging (fMRI) based on blood oxygenation level-dependent (BOLD) contrast has been extensively used for mapping brain function in humans. In particular, after the first observation that spontaneous BOLD fluctuations in the left and right motor cortex are correlated in the absence of a task [4], resting-state fMRI has witnessed an exponential growth of interest. The attractiveness of resting-state fMRI to study brain functional connectivity stems from the potential use as a biomarker for various diseases and from the easy of implementation even for problematic patient populations, since no task is required [5–7].

The BOLD signal is usually measured using gradient-echo (GE) T2*-weighted images due to their large sensitivity to deoxyhaemoglobin variations associated with the hemodynamic response induced by neuronal activity. MRI signal in spin-echo (SE) T2-weighted images is also affected by the local deoxyhaemoglobin content but to a less extent [8–10], with a significant drop in sensitivity (about a factor of 3 at 3T) with respect to its GE counterpart [11–13].

Although less sensitive to BOLD functional signal, SE sequences have been proposed as a potential alternative to obtain increased functional localisation to the capillary bed, because static dephasing effects affecting the extravascular contribution around larger vessels are refocused by the 180° radiofrequency pulse, (see [14] for a recent review). The refocusing pulse is also effective at recovering the signal loss caused by magnetic susceptibility differences at tissue interfaces, suggesting SE fMRI techniques as an attractive solution when e.g. ventromedial frontal and anterior inferior temporal cortex are a main focus of the experiment [12,15,16].

In fact, the superior spatial specificity for the microvasculature of SE fMRI has turned out to be effective only at high field (≥7T), while a significant intravascular contribution from large vessels is still present at low field [14,17]. Recent investigation also showed that signal dropout in regions affected by magnetic field inhomogeneities can be effectively reduced using a dual echo GE EPI as well, with the shorter echo-time yielding a good compromise between reduced signal loss and sufficient fMRI contrast sensitivity in critical regions and the longer echo-time yielding optimal whole brain BOLD sensitivity [18].

However, other studies showed that even at low field the SE BOLD signal has interesting features, apart from spatial specificity or signal drop out recovery. A recent study at 3T showed that the well known saturation of the visual contrast response function [19,20] was more pronounced for the GE BOLD than the SE BOLD signal, allowing the latter an increased ability to distinguish different levels of stimulus-evoked brain activity [21]. Moreover, significant differences in the dynamics of GE and SE BOLD signals were observed at 3T, with SE BOLD responses peaking earlier than GE BOLD responses [22–24]. A reduced “refractoriness” and increased linearity of the SE BOLD signal was also observed in a couple of studies at 4T [25,26]. In these studies, Zhang et al. demonstrated that the BOLD nonlinearity in T2* weighted GE acquisition mainly arises from large-vessel contribution and is responsible of a reduced BOLD amplitude and delayed BOLD onset in response to the second of paired stimuli when the inter-stimulus interval was shorter than 4–6 s [25]. Instead, SE BOLD signals were replicable in response to replicated neuronal activities even at inter-stimulus intervals as short as ~1s, with no significant reduction in amplitude or increase in latency [26]. These findings suggested that the increased linearity and promptness of the SE BOLD signal could have potential benefits for quantitative assessment of the neurovascular coupling relationship and for connectivity studies [21,26].

However, to our best knowledge, there are no reports on the application of spin-echo fMRI for connectivity studies at the most commonly used field strenght of 3T. At high field, where the greatest benefits from SE BOLD are expected, the SAR level due to the refocusing pulse is often a limitation issue imposing sub-optimal repetition rate or brain coverage and only one study demonstrated the detection of SE fMRI resting state networks at 7T, using a specialized EPI sequence to keep acceptable radiofrequency power deposition levels [27].

In this study at 3T we compared GE and SE measurement of resting state functional connectivity in the main networks. In particular we tested i) if the expected drop in sensitivity with SE acquisition is similar to that reported for activation experiments; ii) if SE allows a recover of the functional connectivity signal in regions affected by signal void in GE data; iii) how the two sequences confront each other in the group analysis.

## Methods

### FMRI data acquisition and preprocessing

Resting state BOLD fMRI signals were recorded from 16 right handed healthy subjects (age range between 20 and 31 y; mean age ± st. dev = 23.4 ± 4.3 y; 6 females). Participants were screened by a neurologist/psychiatrist to ensure they had no history of neurological/psychiatric conditions. Other exclusion criteria were chronic medical disorders, current medication, implanted metals, pregnancy and abnormal findings in their structural brain MRI. To avoid any confounding effects of psychoactive substances on the fMRI signal, we also excluded subjects with any drug or alcohol abuse within the previous six months. All subjects gave their written informed consent according to the Declaration of Helsinki (World Medical Association Declaration of Helsinki, 1997) and all procedures were approved by the Ethics Committee for biomedical research of the University G. D'Annunzio—Chieti, Italy. Subjects were instructed to keep their eyes closed and not to engage in structured thoughts.

MRI data were acquired with a 3T Philips Achieva scanner, (Philips Medical Systems, Best, The Netherlands) using a whole-body radiofrequency coil for signal excitation and an 8-channel phased-array head coil for signal reception. Acquisition was performed using gradient-echo (GE) T2*-weighted and spin-echo (SE) T2-weighted echo-planar (EPI) sequences with the following parameters: matrix size 64x64, FOV 230 mm, in-plane voxel size 3.6 mm x 3.6 mm, Sensitivity Encoding (SENSE) factor 1.8 anterior-posterior, flip angle 74°, slice thickness 5 mm and no gap. Echo times were TE = 30 ms for the GE sequences and TE = 75 ms for the SE sequences. Two GE and two SE resting state runs were acquired with a pseudorandomized order across subjects. For each run, 265 functional volumes consisting of 17 transaxial slices were acquired with a TR of 1700 ms.

A high resolution structural volume was also acquired via a 3D fast field echo T1-weighted sequence with the following parameters: 1 mm isotropic voxel size, TR/TE = 8.1/3.7 ms, flip angle = 8°, 160 sections, SENSE factor = 2.

During fMRI, physiological signals related to cardiac and respiratory cycles were registered using a pulse oximeter placed on a finger of the right hand and a pneumatic belt strapped around the upper abdomen, respectively. Cardiac and respiratory data were both sampled at 100 Hz and stored by the scanner’s software in a file for each run.

Resting state fMRI data were analysed using AFNI ([28]; afni.nimh.nih.gov/afni) and custom-written software implemented in Python (http://www.python.org). The first 5 volumes of each functional run were discarded to allow T1 equilibration of the MR signal. Initial preprocessing steps included despiking (AFNI’s “3dDespike”) to remove transient signal spikes from the EPI time series, RETROICOR [29] to remove signal fluctuations time locked to cardiac and respiratory cycles, slice scan time correction and motion correction. RETROICOR was performed regressing out physiological noise components from the EPI time series, considering 8 slice-wise regressors generated by AFNI’s “RetroTS.m” code using the logged cardiac and respiratory waveforms as input. Motion correction was done by rigid body registration of EPI images to a base image represented by the first volume of the first run, separately for GE and SE. A summary statistic of motion was defined as the root mean square (RMS) of the six realignment parameters (three translations and three rotations), in order to inspect for runs affected by excessive motion. One GE run and one SE run exceeded a root mean square (RMS) movement of 1.5 mm and were discarded from further analysis.

The preprocessed functional time series were coregistered with the corresponding structural data set using an affine transformation and resampled to a 3mmx3mmx3mm grid. The motion correction and coregistration transformations were applied at once to the EPI images to prevent multiple resampling steps.

Then, additional preprocessing was performed using the ANATICOR method [30] to remove further physiological and hardware related confounds. Individual masks of large ventricles and white matter to be used in this approach were obtained from the structural scans segmentation using FreeSurfer (http://surfer.nmr.mgh.harvard.edu). The white matter mask was eroded slightly (one functional voxel) to prevent partial volume effects that might include signal from gray matter voxels in the mask. This step was not performed for the CSF mask, since with the functional voxel size used in this study the eroded CSF mask would not contain enough voxels in most subjects. Then, for each run, a global nuisance regressor was obtained extracting the EPI average time course within the ventricle mask and local nuisance regressors were obtained calculating for each gray matter voxel the average signal time course for all white matter voxels within a 3 cm radius [30]. These nuisance regressors and the six regressors derived from motion parameters were removed from the EPI timeseries using AFNI’s @ANATICOR.

Further preprocessing steps to prepare the data for functional connectivity analysis included spatial normalization into the Talairach space using an affine transformation, spatial smoothing with an isotropic gaussian kernel (full width at half maximum = 6 mm), scaling to a common mean and temporal band-pass filtering in the frequency band considered of interest for resting state fMRI (0.01 < f < 0.1 Hz). Finally, the two runs for each sequence were concatenated resulting in voxel time courses with 520 time points. Prior to functional connectivity analysis, a volume censoring technique [31,32] was used to reduce motion related artifacts in resting state correlations calculations potentially not accounted for by spatial registration and regression of motion parameters. Briefly, the framewise displacement (FD) and the root mean square value of the differentiated BOLD timeseries (DVARS) within a whole brain spatial mask were used as quality control measures to guide the censoring procedure [31]; [33]. Here FD > 0.2 mm and DVARS > 0.3% ΔBOLD were used to remove frames likely affected by excessive movement [34]. Two volumes before and two volumes after the flagged volumes were removed as well to account for spin history effects and possible temporal spread of motion related artifactual signal.

The run-averaged DVARS and FD metrics and the correlations between DVARS-FD timeseries were also compared for GE and SE in order to characterize a potential difference in motion sensitivity of the two sequences.

### Functional connectivity analysis

First, seed-based resting state connectivity maps were created for each subject and sequence calculating the Pearson correlation coefficient (r-value) between the seed time series and the time series at each voxel. The time series at each seed was derived averaging the time courses of voxels inside a sphere with 6mm radius centered at the seed’s coordinates. Five seeds were chosen compatibly with the cited references to reflect established resting state networks (see Table 1): 1) the posterior cingulate cortex [35] for the default mode network (DMN), 2) the left inferior parietal lobule [36] for the executive control network (ECN), 3) the left anterior cingulate cortex [37] for the salience network (SN), 4) the left inferior parietal sulcus [38] for the dorsal attention network (DAN), 5) the left primary motor cortex [39] for the sensorimotor network (SMN).

**Table 1:** Random effects group analysis: Talairach coordinates (peak voxel) of the areas significantly connected to the seed for GE and SE.

		GE			SE			Mean coordinates		
Node	Network	X	Y	Z	X	Y	Z	X	Y	Z
**L_M1**	Motor							−33	−28	51
SMA	Motor	−3	−25	50	−1	−23	51	−2	−24	50.5
R_M1	Motor	32	−27	48	28	−31	49	30	−29	48.5
**L_IPL**	ECN							−41	−59	37
R_IPL	ECN	41	−64	39	39	-62	40	40	−63	39.5
R_MidFG	ECN	46	14	38	47	16	38	46.5	15	38
L_MidFG	ECN	−44	13	38	−44	10	37	−44	11.5	37.5
L_MedFG	ECN	−3	25	47	−2	24	45	−2.5	24.5	46
**L_ACC**	Salience							−3	32	24
R_aI	Salience	−32	16	−4	−32	12	−3	−32	14	−3.5
L_aI	Salience	32	16	−4	34	12	−2	33	14	−3
R_SupFG	Salience	24	36	35	23	39	34	23.5	37.5	34.5
L_SupFG	Salience	−28	39	31	−24	43	23	−26	41	27
**L_IPS**	DAN							−32	−47	45
R_IPS	DAN	37	−46	48	35	-46	48	36	−46	48
R_Fef	DAN	26	−7	48	25	−7	48	25.5	−7	48
L_Fef	DAN	−21	−9	51	−20	−5	48	−20.5	−7	49.5
dACC	DAN	5	3	46	5	3	46	5	3	46
**PCC**	DMN							−3.6	−58.1	27.5
R_AG	DMN	50	−58	25	53	−54	23	51.5	−56	24
L_AG	DMN	−46	−62	26	−47	−64	25	−46.5	−63	25.5
MedFG	DMN	7	41	22	7	45	28	7	43	25
MedFG	DMN	−2	54	3	5	55	2	1.5	54.5	2.5
MedFG_vent	DMN				−3	42	−8	-3	42	−8
L_Hippocampus	DMN	−23	−34	−9	−26	−34	−9	−24.5	−34	−9
R_Hippocampus	DMN	24	−34	−9	25	−34	−9	24.5	−34	−9
L_MidTempG	DMN	−57	−10	−8	−60	−10	−9	−58.5	−10	−8.5
R_MidTempG	DMN	60	−10	−10	60	−12	−10	60	−11	−10

The regions chosen as seeds for the investigated networks are represented in bold. The coordinates used to define the spherical nodes (mean coordinates between GE and SE) for the comparison of functional connectivity strenght for the two sequences are also reported.

L_M1 = Left Primary Motor Area, SMA = Supplementary Motor Area, L_IPL = Left Inferior Parietal Lobule, R_IPL = Right Inferior Parietal Lobule, R_MidFG = Right Middle Frontal Gyrus, L_MidFG = Left Middle Frontal Gyrus, L_ACC = Left Anterior Cingulate Cortex, R_aI = Right Anterior Insula, L_aI = Left Anterior Insula, R_SupFG = Right Superior Frontal Gyrus, L_SupFG = Left Superior Frontal Gyrus, L_IPS = Left Intraparietal Sulcus, R_IPS = Right Intraparietal Sulcus, L_Fef = Left Frontal eye field, R_Fef = Right Frontal eye field, dACC = dorsal Anterior Cingulate Cortex, PCC = Posterior Cingulate Cortex, R_AG = Right Angular Gyrus, L_AG = Left Angular Gyrus, MedFG = Medial Frontal Gyrus, MedFG_vent = Medial Frontal Gyrus ventral, R_Hyppocampus = Right Hyppocampus, L_Hyppocampus = Left Hyppocampus, L_MidTempG = Left Middle Temporal Gyrus, R_MidTempG = Right Middle Temporal Gyrus.

Then, individual correlation maps were converted using z-Fisher transformation (z = atanh(r), where r is the correlation coefficient) to approach a normal distribution before entering the group analysis. A one-sample t-test was performed on the z-Fisher maps to obtain group statistical functional connectivity maps for GE and SE.

Group statistical maps were thresholded at p<0.05 (corrected for multiple comparisons) using a cluster-size thresholding algorithm [40] based on Monte Carlo simulations (AFNI’s AlphaSim). A threshold of p<0.001 at the voxel level and a minimum cluster size of ten voxels yielded the required alpha level of p<0.05, corrected for multiple comparisons.

For each network, a number of nodes were defined considering the clusters of voxels showing a significant (p< 0.05 corrected) correlation with the seed in the corresponding group connectivity map. The Talairach coordinates of the peak voxel of each cluster were obtained separately for the GE and SE sequences and the corresponding anatomical structures were identified using the AFNI atlas. Then, a common region of interest for GE and SE was defined for each node in order to compare the BOLD timecourse for the two sequences in the same group of voxels. These common nodes were obtained by drawing a sphere of 6 mm of radius centered at a voxel with average coordinates between those of the previously determined GE and SE peak voxels. This procedure for the nodes definition was followed to reduce a potential bias toward one of the two sequences. For the same reason we did not use independent coordinates from the literature, since all the existing resting state connectivity studies at 3T are performed with the GE sequence. In order to compare the connectivity strenght as measured with the two sequences, the mean correlation coefficient from voxels inside the spherical region associated to each node was calculated for the investigated seed-based correlation maps and averaged across subjects. The mean t-values for each node was also extracted from group connectivity maps to compare the sensitivity of the two sequences at the group level.

Finally, to inspect the temporal and frequency characteristics of GE and SE BOLD signal fluctuations, the power spectrum density (PSD) of the EPI timeseries extracted from each seed was estimated using the Welch method (window of 100 data points with an overlap of 50 data points). Individual PSDs were normalized to 1 (integral normalization) and averaged across subjects.

## Results

The run averaged RMS, FD and DVARS quality measures yielded similar values for GE (RMS: 0.46 ± 0.39 mm; FD = 0.08 ± 0.03 mm; DVARS = 0.20 ± 0.04% ΔBOLD: mean values across subjects ± s.d.) and SE (RMS: 0.41 ± 0.44 mm; FD = 0.08 ± 0.02 mm; DVARS = 0.21 ± 0.05% ΔBOLD: mean values across subjects ± s.d.). The correlation between DVARS and FD timeseries was also comparable for GE and SE (0.09 ± 0.06 and 0.10 ± 0.07 respectively, mean values across subjects ± s.d.). On average, 7.3 ± 11.4 volumes per run were censored for GE and 9.1 ± 11.7 volumes for SE.

**Fig 1:**
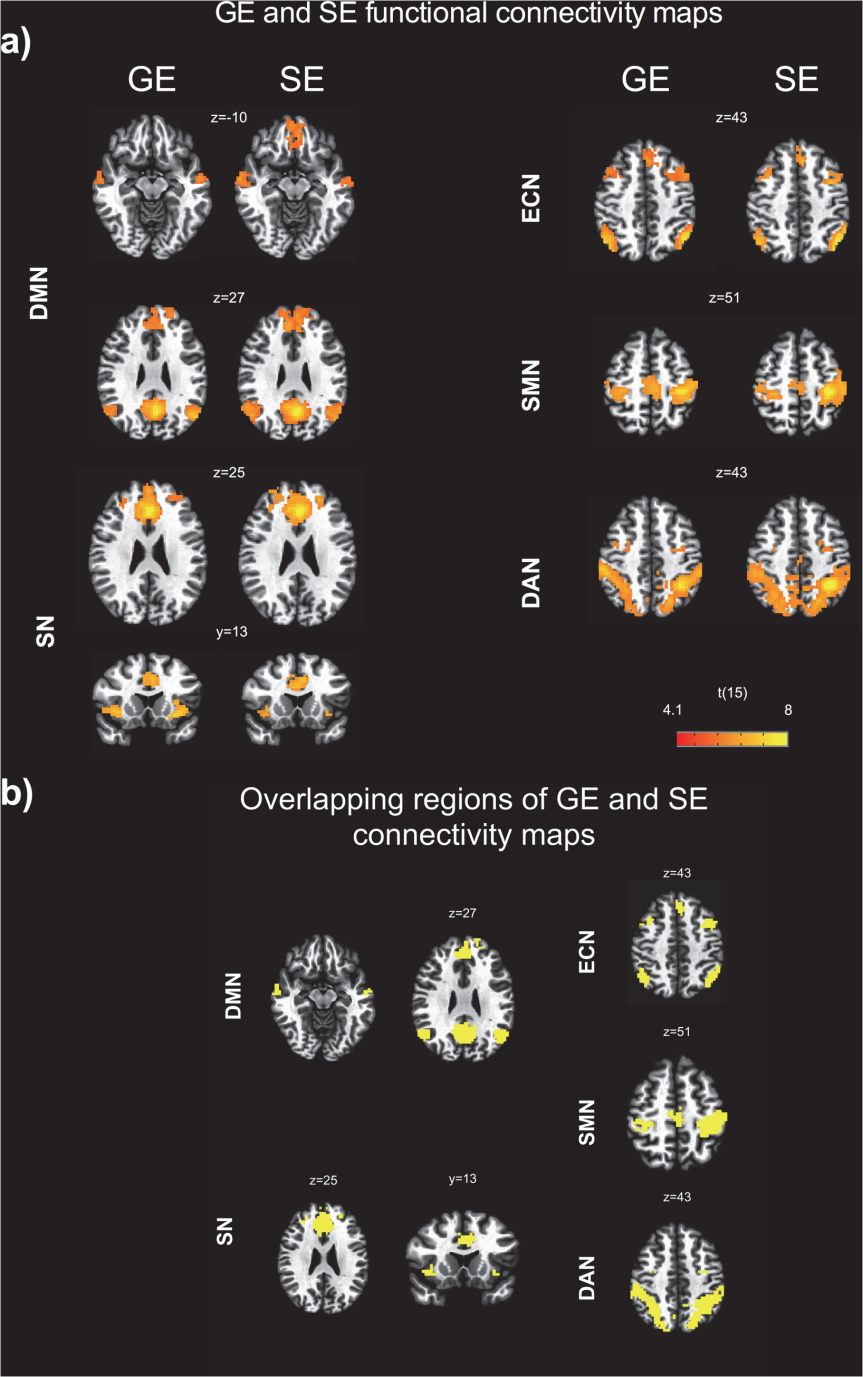
GE and SE functional connectivity maps. a) seed based connectivity maps for gradient-echo (GE) and spin-echo (SE) obtained from the random effects group analysis showing the following resting state networks: default mode network (DMN), executive control network (ECN), salience network (SN), dorsal attention network (DAN), sensorimotor network (SMN). The group statistical maps were thresholded at p < 0.05, corrected for multiple comparisons using a cluster-size algorithm, and superimposed on the Talairach template; b) overlapping regions of GE and SE connectivity maps. Images are displayed using the radiological convention., i.e., right is left, left is right.

The group analysis showed significant functional connectivity maps in both GE and SE data (Fig. 1A).

The spatial patterns were largely overlapping for the two sequences (Fig. 1B), except for the ventromedial prefrontal cortex of the DMN that did not show a significant connectivity with the seed in the GE acquisition.

The nodes identified on the group connectivity maps are reported in Table 1, together with the Talairach coordinates of the peak voxels.

**Fig 2:**
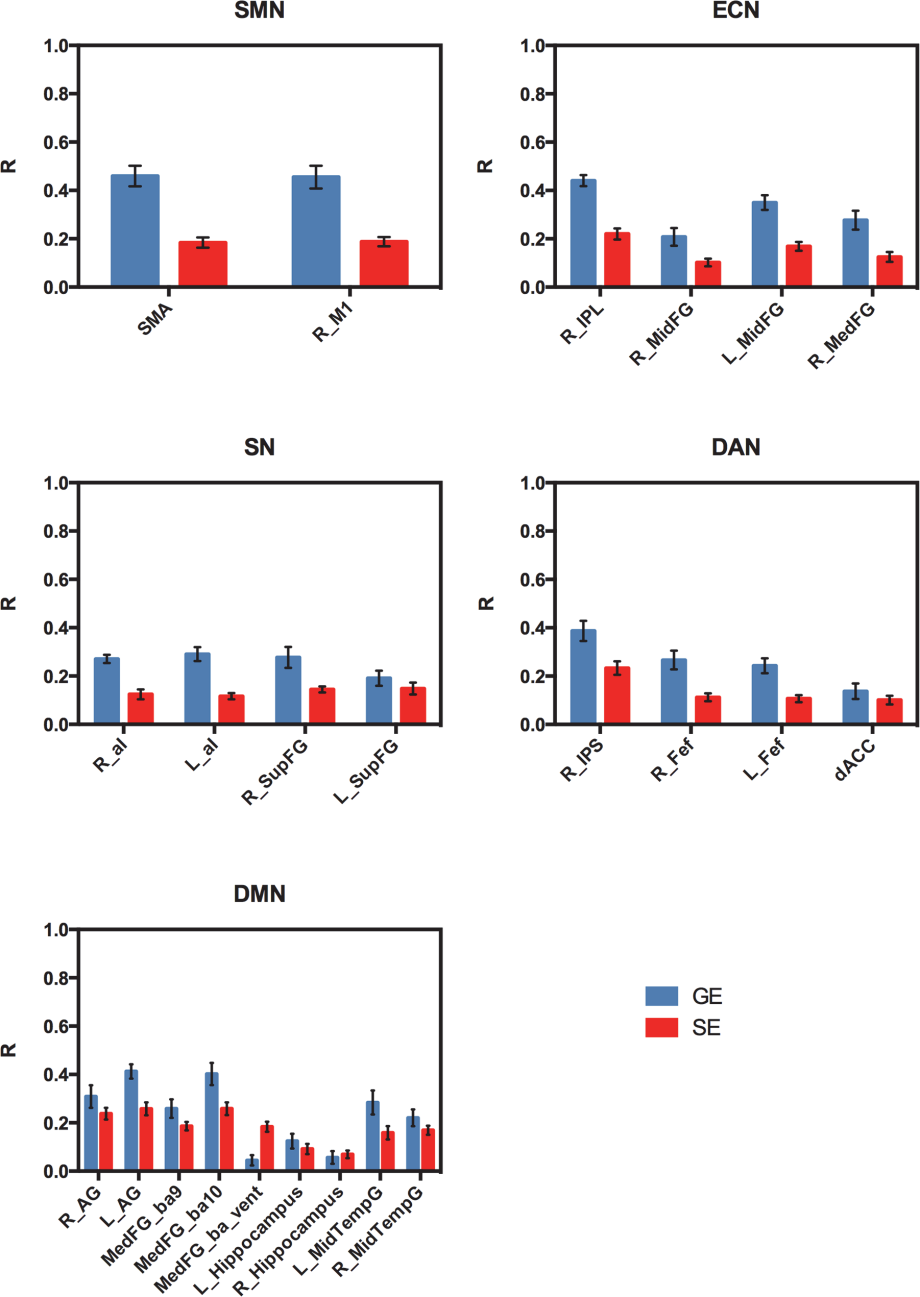
Correlation values for the different node-seed functional connections as measured with GE and SE. The r-values were first averaged across voxels inside a node on individual correlation maps and then averaged across subjects. Error bars are standard errors.

**Fig 3:**
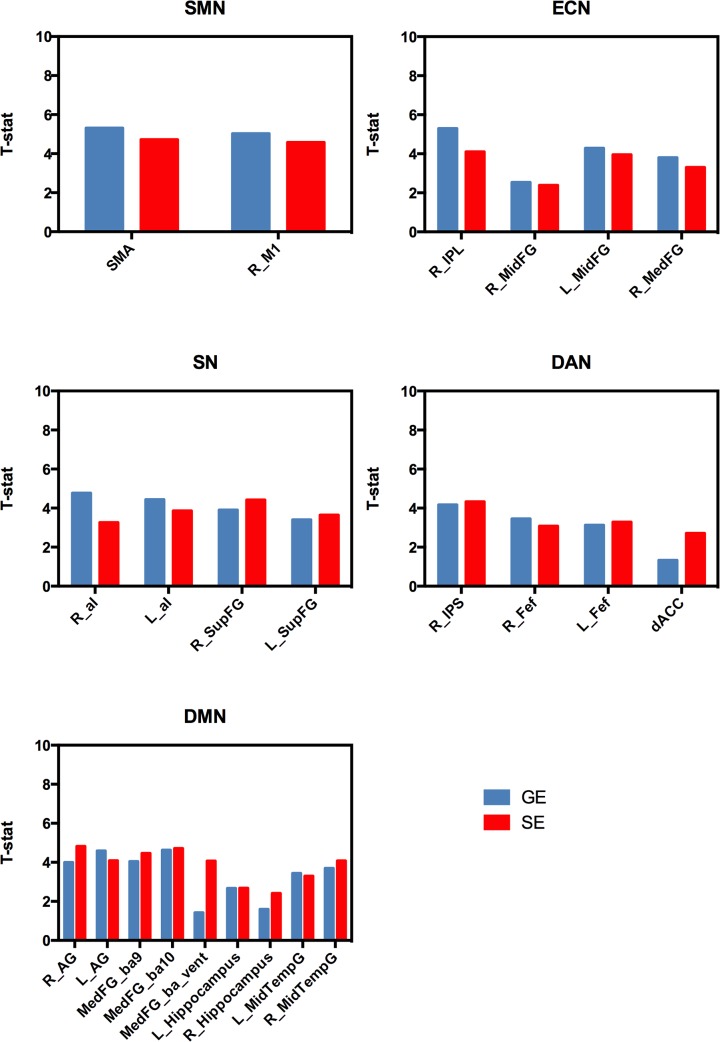
Statistical significance of the group connectivity maps in the different nodes. One sample t-values were obtained averaging t-values across voxels inside each node in the random effects group connectivity maps. Note the similar t-values for GE and SE despite the reduced sensitivity of the latter.

The mean correlation values across subjects for the different node-seed connections are shown in Fig. 2. As expected, the larger sensitivity of GE acquisition to the BOLD signal fluctuations yielded larger correlation values with respect to SE acquisition (about a factor of 2.5 for SMN, 1.9 for ECN, 1.9 for SN, 1.6 for DAN and 1.2 for DMN). However, SE correlation coefficients exhibited a lower intersubject variability as shown by the smaller standard errors. As a consequence, the statistical significance in the group level connectivity maps was comparable for the two sequences (the one sample t-test values are shown in Fig. 3).

**Fig 4:**
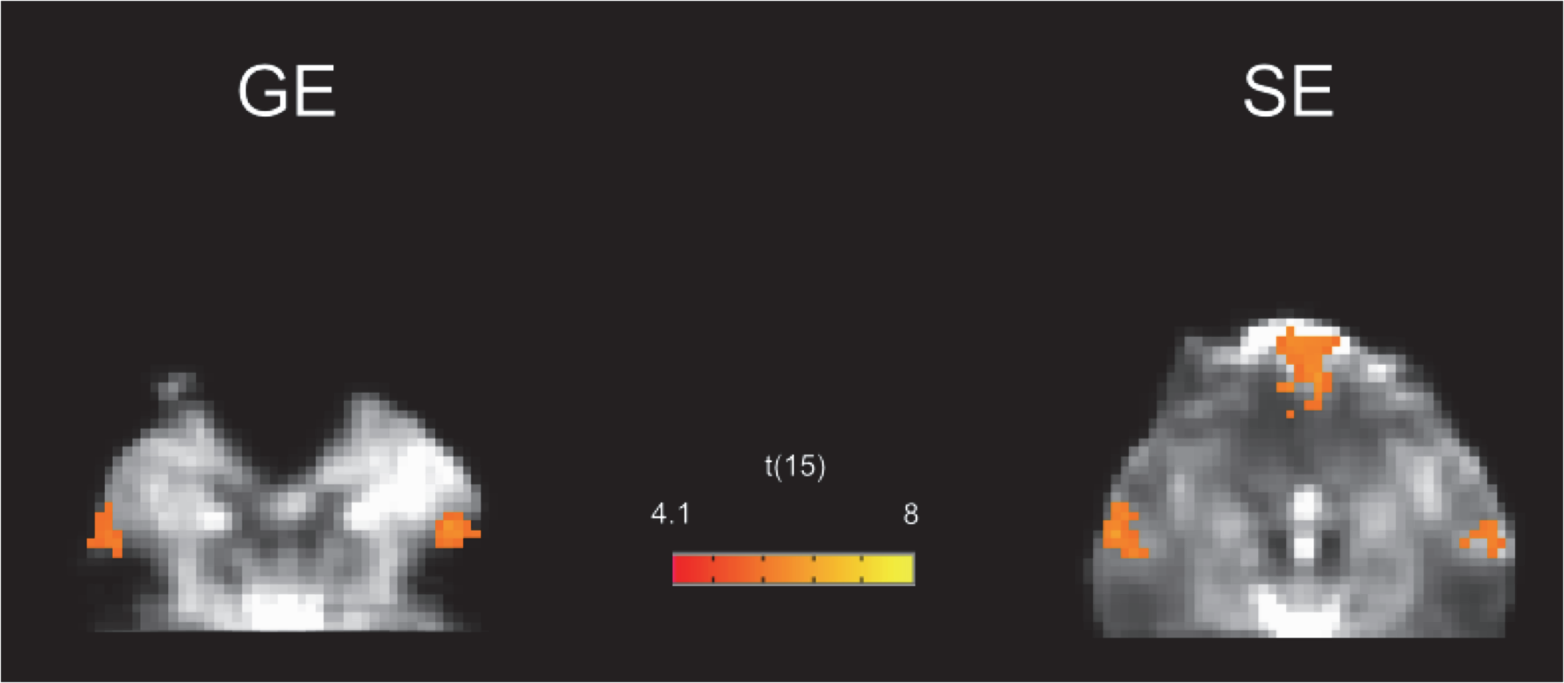
GE and SE group connectivity maps for the default mode network in the inferior brain regions. The map is superimposed on the Talairach transformed EPI images of one subject. The signal void is clearly visible in the GE images, with the corresponding loss of functional contrast in the ventromedial prefrontal node. Both raw signal and functional contrast are instead recovered in SE images.

A remarkable exception was the ventromedial prefrontal node of DMN, that showed larger t-values and r-values for SE. In this region, the GE-EPI functional images showed the well known signal voiding artifact, whereas no susceptibility-induced signal voiding was found in the SE-EPI images (Fig. 4).

**Fig 5:**
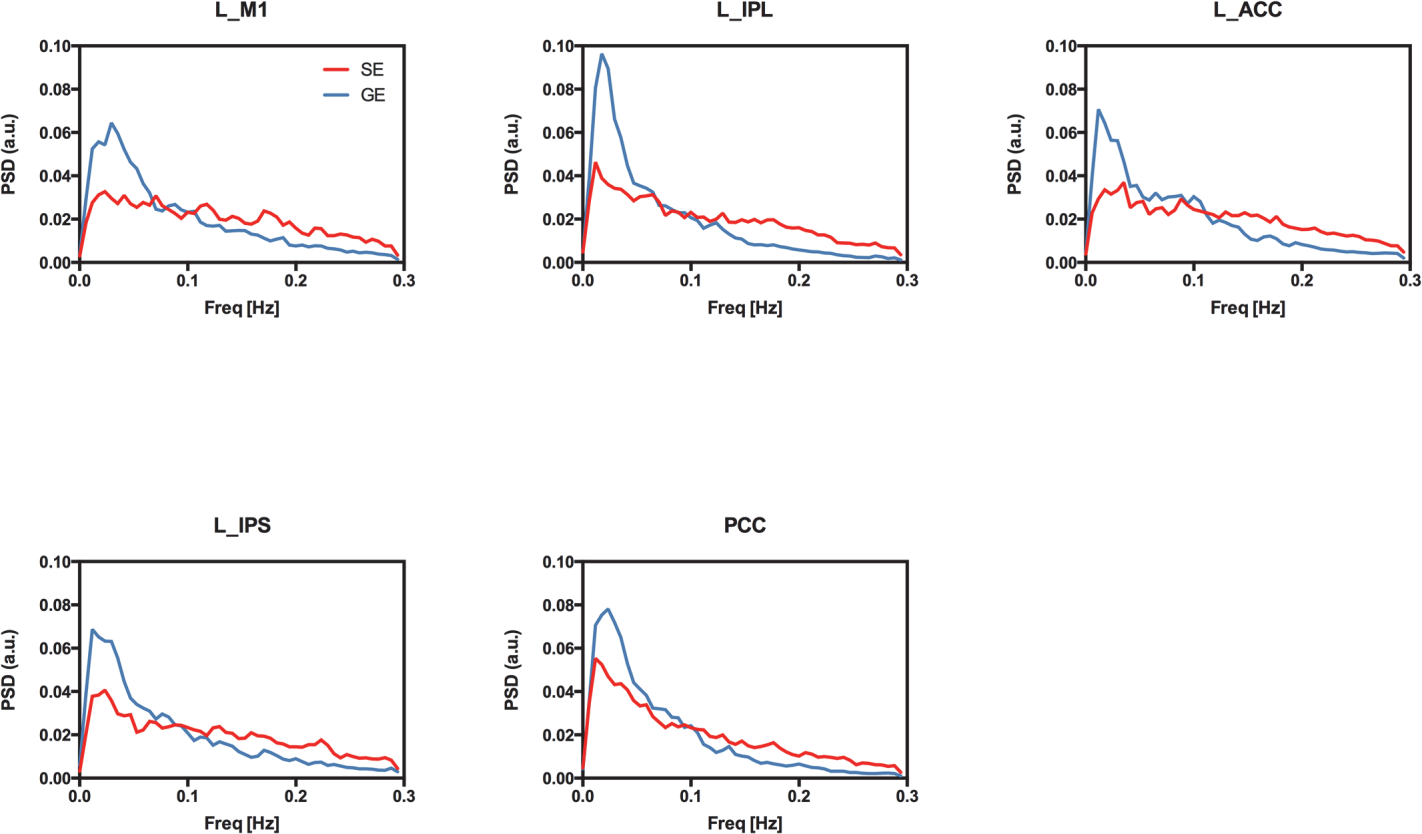
Power spectra of GE and SE EPI timeseries extracted from the considered seed regions. The individual power spectra were first normalized to 1 (integral normalization) and then averaged across subjects. Note the faster decay of the power of GE BOLD oscillations at increasing frequencies.

The power spectrum density of the seed time courses showed a different frequency distribution of the power of resting state oscillations, with the magnitude of GE BOLD spectrum decaying faster than that of SE BOLD (Fig. 5).

## Discussion

In this study we compared resting state BOLD connectivity using spin-echo and gradient-echo EPI sequences at 3T. To our best knowledge, this is the first observation of resting state networks using spin-echo fMRI at low field. The seed based group functional connectivity maps obtained with the two sequences were largely overlapping. However some differences in the spatial pattern were observed for the default mode network, that showed a significant functional connectivity of the ventromedial frontal node for SE only.

Overall, the larger BOLD sensitivity of GE yielded expected larger correlation values than SE. This difference in correlation values exhibited some variation across networks and was larger for the somatomotor, salience and executive control networks (about a factor of 2.1) than for the dorsal attention and default mode networks (about a factor of 1.4). Moreover, in the random-effect group analysis the reduced sensitivity of SE was counterbalanced by a reduced intersubject variability, resulting in group level statistical significance comparable to GE.

The increased functional contrast of SE that we observed in the ventromedial prefrontal node of DMN can be explained by the macroscopic magnetic field inhomogeneities affecting this region that create signal loss in GE images. The signal void was restored in SE images, as clearly shown by the comparison of the EPI image intensity for the two sequences. This finding is in line with those of previous studies on task related activity, where the spin-echo method performed better than gradient-echo in similar problematic regions affected by signal drop-out [12,16,41]. Although other studies showed that optimized gradient-echo or dual gradient-echo sequences [15,18] can restore the signal loss in critical regions yielding the same functional contrast as SE fMRI, this was not tested for resting state connectivity.

The larger correlation values exhibited by GE in most cortical areas is to be expected due to the contribution of static dephasing effects around large vessels to the GE BOLD signal fluctuations. Previous studies on task related activity showed a three-fold or higher GE vs SE sensitivity at 3T [12,13]. Noteworthy, our data show that for resting state connectivity the difference in sensitivity between the two sequences is less than what reported for activation studies, with DMN showing GE correlation values only 1.2 larger than corresponding SE values.

A possible explanation of this behaviour can be related to the increased linearity and promptness of the SE BOLD signal demonstrated in previous studies [22–24,26]. It can be argued that these features allow the SE fMRI signal to follow the intrinsic ongoing brain activity more accurately than GE, partly compensating for the reduced amplitude of the T2 weighted BOLD oscillations. In task related activation during block paradigms, as those used in most of previous reports on SE, these temporal aspects and the sluggishness of the haemodynamic response are likely to play a minor role and the increased GE vs SE sensitivity is mainly the result of the larger T2* weighted BOLD signal change.

The differences we observed in the frequency characteristics of GE and SE BOLD signals also point to a relatively increased high frequency oscillations in the latter, possibly resulting from the reduced refractoriness of the microvascular compartment [26]. Interestingly, the power spectrum of resting state SE BOLD signal in our data resembles that of spontaneous fluctuations of cerebral blood volume (CBV) weighted signal reported in a recent study using the vascular space occupancy (VASO) technique [42]. Further investigation is needed to clarify if this similarity also reflect a common physiological mechanism contributing to the oscillations of the two signals.

Another possible explanation for the present results is that the hemodynamic response can vary among regions of the brain with respect to both linearity and shape [43–46]. Although part of this variation can be neuronal in origin, it has also been shown that vascular contributions are important [46]. In particular, a different macrovascular architecture across brain regions is expected to introduce spurious differences and delays among the associated GE BOLD time courses (see [47] for a review), due to a different contribution of static dephasing effects around large vessels. These vascular related differences in the hemodynamic responses can lower the correlation between GE BOLD timecourses from two brain regions, despite the associated intrinsic neuronal activities fluctuate coherently. In contrast, the microvascular architecture is more uniform throughout the cortex, suggesting that SE BOLD timecourses should be affected by this confound to a less extent.

The effect of the vasculature would also explain the lower intersubject variability of SE correlation values. Indeed, while the random orientation of capillary and venules in tissue results in a rather uniform microvascular architecture across individuals and brain regions, this is not true for larger vessels. For example surface veins are oriented along one direction, according to individual and local anatomy. It is well known from biophysical models of the BOLD signal that the orientation of blood vessels relative to the main magnetic field directly influences the magnitude of the static dephasing effects, with the largest effect expected for 90° oriented vessels [17,48–50].

In our results, the reduced inter subject variability of the SE response improves the random effects analysis at the point that the SE and GE group connectivity maps show similar statistical significance.

Interestingly, a similar effect has been observed in activation studies comparing GE BOLD with functional perfusion based on arterial spin labelling (ASL) [51]. Aguirre et al. showed that, despite GE BOLD benefits from a larger signal to noise within subjects, the perfusion method provided a more robust result across subjects. This finding could be explained by the authors considering that the evoked signal magnitude was more consistent across subjects for perfusion compared to GE BOLD, compensating for the reduced signal to noise of arterial spin labeling. This similarity between SE BOLD and arterial spin labeling is not surprising, given that both techniques are less sensitive to large vessel confounds with respect to GE BOLD, adding support to the hypothesis that macrovascular effects can increase intersubject variability in functional measurements.

In a recent study combining simultaneous GE BOLD and ASL acquisition with magnetic resonance angiography, it has also been shown that the coupling between resting-state GE BOLD and ASL signal fluctuations is highly variable in the brain but is significantly larger in voxels with a reduced macrovascular volume fraction [52]. The strongest GE BOLD-ASL coupling was observed in regions corresponding to major nodes of the default mode and dorsal attention networks, suggesting a reduced macrovascular contribution to the BOLD signal in these areas [52]. Noteworthy, in agreement with these previous findings, the default mode and dorsal attention networks are the two networks exhibiting the lowest GE/SE ratio in our data. It can hence be speculated that the variable sensitivity difference between GE and SE sequences that we observed across networks might reflect different macrovascular contributions to BOLD signal fluctuations in these systems.

Regarding the functional connectivity analysis, a seed based approach was used in this study rather than independent component analysis (ICA). ICA decomposes fMRI data into a set of spatially independent maps and corresponding time courses and has the benefit of being a data driven technique. However, although similar resting state networks are obtained with the two analysis techniques [53], recent evidence suggested that the seed based approach offers a larger reliability and reproducibility of connectivity measures at the individual subject level [54]. The interpretation of seed based correlations values as functional connection strenght is also straightforward and easy to relate to measures of e.g. white matter integrity or structural connectivity [55,56]. Furthermore, correlation values between time courses can be used as input for other metrics of functional networks, as those obtained with graph analysis [57].

From a methodological point of view, a potential improvement to be considered in future studies is the use of a combined dual echo EPI sequence with simultaneous acquisition of GE and SE images [16,58] that would allow a finer comparison of the two signals. However in resting state measurements, within subject inter-run variability is expected to be reasonably low and is likely cancelled out at group level. Another possible source of criticism is that the long readout in standard EPI acquisition actually introduces a certain T2* weighting also in SE EPI. To limit this effect we used a large bandwidth and the SENSE parallel imaging technique [59] which allows a faster encoding of the MRI image. Noteworthy, the partial T2* weighting of SE EPI would render the corresponding fMRI signal more similar to the GE fMRI signal, rather than artificially introducing differences that could enhance or bias the comparison between the two sequences.

Another factor that could bias this comparison is a possible difference in sensitivity of the two sequences with regard to movement, a feature that has been shown particularly relevant for resting state functional connectivity [31,32]. Here, the motion-related quality control measures did not show significant differences between the two sequences, suggesting that this should be a minor issue for the present comparison.

Furthermore, it cannot be excluded that the larger variability of GE correlation values could reflect true population heterogeneity in functional connectivity that is not recognized by SE. In this regard future studies with simultaneous EEG/fMRI measurements or combining structural and functional connectivity DTI are needed to better clarify this aspect.

Finally, a further limitation of the study can be the small sample size. Future studies with larger sample sizes should be performed to replicate and confirm the present results.

In conclusion, while previous studies at low field compared GE and SE for task-evoked BOLD responses, this is the first study comparing functional connectivity measurements obtained with the two sequences at 3T. Main results showed that, within subject, GE BOLD sensitivity for resting state connectivity is larger with respect to SE BOLD, but to a less extent than what reported for task related activation studies. In contrast, SE BOLD sensitivity was larger in the ventral node of DMN, a region suffering from susceptibility signal voiding in GE acquisition. Moreover, SE BOLD data showed a reduced across-subject variability, resulting in similar statistical connectivity maps at group level for the two sequences. These findings show that, despite the lower within subject sensitivity, SE BOLD offers the same sensitivity of GE BOLD when addressing hypotheses to the population level, with the added benefit of recovered functional connectivity signal from regions affected by magnetic field inhomogeneity. Future studies should clarify if the reduced intersubject variability of SE data can help to distinguish subtle variations of functional connectivity across different populations and/or treatments when vascular confounds can be a critical issue.

## References

[1] Bandettini PA, Wong EC, Hinks RS, Tikofsky RS, Hyde JS (1992) Time course EPI of human brain function during task activation. Magn Reson Med 25: 390–397. 1614324 10.1002/mrm.1910250220

[2] Kwong KK, Belliveau JW, Chesler DA, Goldberg IE, Weisskoff RM, et al (1992) Dynamic magnetic resonance imaging of human brain activity during primary sensory stimulation. Proceedings of the National Academy of Sciences of the United States of America 89: 5675–5679. 10.1073/pnas.89.12.5675 1608978 PMC49355

[3] Ogawa S, Tank DW, Menon R, Ellermann JM, Kim SG, et al (1992) Intrinsic signal changes accompanying sensory stimulation: Functional brain mapping with magnetic resonance imaging. Proceedings of the National Academy of Sciences of the United States of America 89: 5951–5955. 10.1073/pnas.89.12.5675 1631079 PMC402116

[4] Biswal B, Zerrin Yetkin F, Haughton VM, Hyde JS (1995) Functional connectivity in the motor cortex of resting human brain using echo‐planar mri. Magnetic Resonance in Medicine 34: 537–541. 10.1002/mrm.1910340409 8524021

[5] Fox MD, Raichle ME (2007) Spontaneous fluctuations in brain activity observed with functional magnetic resonance imaging. Nat Rev Neurosci 8: 700–711. 10.1038/nrn2201 17704812

[6] Fox MD, Greicius M (2010) Clinical applications of resting state functional connectivity. Front Syst Neurosci 4: 19 10.3389/fnsys.2010.00019 20592951 PMC2893721

[7] Brier MR, Thomas JB, Ances BM (2014) Network Dysfunction in Alzheimer's Disease: Refining the Disconnection Hypothesis. Brain Connectivity 4: 299–311. 10.1089/brain.2014.0236 24796856 PMC4064730

[8] Bandettini PA, Wong EC, Jesmanowicz A, Hinks RS, Hyde JS (1994) Spin-echo and gradient-echo EPI of human brain activation using BOLD contrast: a comparative study at 1.5 T. NMR Biomed 7: 12–20. 8068520 10.1002/nbm.1940070104

[9] Harmer J, Sanchez-Panchuelo RM, Bowtell R, Francis ST (2012) Spatial location and strength of BOLD activation in high-spatial-resolution fMRI of the motor cortex: a comparison of spin echo and gradient echo fMRI at 7 T. NMR Biomed 25: 717–725. 10.1002/nbm.1783 21948326

[10] Jones RA, Schirmer T, Lipinski B, Elbel GK, Auer DP (1998) Signal undershoots following visual stimulation: A comparison of gradient and spin-echo bold sequences. Magnetic Resonance in Medicine 40: 112–118. 9660561 10.1002/mrm.1910400116

[11] Jochimsen TH, Norris DG, Mildner T, M ller HE (2004) Quantifying the intra- and extravascular contributions to spin-echo fMRI at 3 T. Magnetic Resonance in Medicine 52: 724–732. 10.1002/mrm.20221 15389950

[12] Norris DG, Zysset S, Mildner T, Wiggins CJ (2002) An investigation of the value of spin-echo-based fMRI using a Stroop color-word matching task and EPI at 3 T. NeuroImage 15: 719–726. 10.1006/nimg.2001.1005 11848715

[13] Parkes LM, Schwarzbach JV, Bouts AA, Deckers RHR, Pullens P, et al (2005) Quantifying the spatial resolution of the gradient echo and spin echo BOLD response at 3 Tesla. Magn Reson Med 54: 1465–1472. 10.1002/mrm.20712 16276507

[14] Norris DG (2012) Spin-echo fMRI: The poor relation? NeuroImage 62: 1109–1115. 10.1016/j.neuroimage.2012.01.003 22245351

[15] Schmidt CF, Boesiger P, Ishai A (2005) Comparison of fMRI activation as measured with gradient- and spin-echo EPI during visual perception. NeuroImage 26: 852–859. 10.1016/j.neuroimage.2005.02.043 15955495

[16] Schwarzbauer C, Mildner T, Heinke W, Brett M, Deichmann R (2010) Dual echo EPI—the method of choice for fMRI in the presence of magnetic field inhomogeneities? NeuroImage 49: 316–326. 10.1016/j.neuroimage.2009.08.032 19699805

[17] Uludağ K, Müller-Bierl B, Uğurbil K (2009) An integrative model for neuronal activity-induced signal changes for gradient and spin echo functional imaging. NeuroImage 48: 150–165. 10.1016/j.neuroimage.2009.05.051 19481163

[18] Halai AD, Welbourne SR, Embleton K, Parkes LM (2014) A comparison of dual gradient-echo and spin-echo fMRI of the inferior temporal lobe. Hum Brain Mapp 35: 4118–4128. 10.1002/hbm.22463 24677506 PMC6869502

[19] Boynton GM, Engel SA, Glover GH, Heeger DJ (1996) Linear systems analysis of functional magnetic resonance imaging in human V1. J Neurosci 16: 4207–4221. 8753882 10.1523/JNEUROSCI.16-13-04207.1996 PMC6579007

[20] Park JC, Zhang X, Ferrera J, Hirsch J, Hood DC (2008) Comparison of contrast-response functions from multifocal visual-evoked potentials (mfVEPs) and functional MRI responses. J Vis 8: 8.1–.12. 10.1167/8.10.8 PMC2987574 19146350

[21] Chiacchiaretta P, Romani GL, Ferretti A (2013) Sensitivity of BOLD response to increasing visual contrast: spin echo versus gradient echo EPI. NeuroImage 82: 35–43. 10.1016/j.neuroimage.2013.05.069 23707589

[22] Hulvershorn J, Bloy L, Gualtieri EE, Leigh JS, Elliott MA (2005) Spatial sensitivity and temporal response of spin echo and gradient echo bold contrast at 3 T using peak hemodynamic activation time. NeuroImage 24: 216–223. 10.1016/j.neuroimage.2004.09.033 15588613

[23] Hulvershorn J, Bloy L, Gualtieri EE, Redmann CP, Leigh JS, et al (2005) Temporal resolving power of spin echo and gradient echo fMRI at 3T with apparent diffusion coefficient compartmentalization. Hum Brain Mapp 25: 247–258. 15849715 10.1002/hbm.20094 PMC6871739

[24] Kohno S, Sawamoto N, Urayama SI, Aso T, Aso K, et al (2009) Water-diffusion slowdown in the human visual cortex on visual stimulation precedes vascular responses. J Cereb Blood Flow Metab 29: 1197–1207. 10.1038/jcbfm.2009.45 19384332

[25] Zhang N, Zhu X-H, Chen W (2008) Investigating the source of BOLD nonlinearity in human visual cortex in response to paired visual stimuli. NeuroImage 43: 204–212. 10.1016/j.neuroimage.2008.06.033 18657623 PMC2614294

[26] Zhang N, Yacoub E, Zhu X-H, Uğurbil K, Chen W (2009) Linearity of blood-oxygenation-level dependent signal at microvasculature. NeuroImage 48: 313–318. 10.1016/j.neuroimage.2009.06.071 19580875 PMC2760467

[27] Koopmans PJ, Boyacioğlu R, Barth M, Norris DG (2012) Whole brain, high resolution spin-echo resting state fMRI using PINS multiplexing at 7 T. NeuroImage 62: 1939–1946. 10.1016/j.neuroimage.2012.05.080 22683385

[28] Cox RW (1996) AFNI: software for analysis and visualization of functional magnetic resonance neuroimages. Comput Biomed Res 29: 162–173. 8812068 10.1006/cbmr.1996.0014

[29] Glover GH, Li TQ, Ress D (2000) Image‐based method for retrospective correction of physiological motion effects in fMRI: RETROICOR. Magnetic Resonance in Medicine. 10.1002/1522-2594(200007)44:1<162::aid-mrm23>3.0.co;2-e 10893535

[30] Jo HJ, Saad ZS, Simmons WK, Milbury LA, Cox RW (2010) Mapping sources of correlation in resting state FMRI, with artifact detection and removal. NeuroImage 52: 571–582. 10.1016/j.neuroimage.2010.04.246 20420926 PMC2897154

[31] Power JD, Barnes KA, Snyder AZ, Schlaggar BL, Petersen SE (2012) Spurious but systematic correlations in functional connectivity MRI networks arise from subject motion. NeuroImage 59: 2142–2154. 10.1016/j.neuroimage.2011.10.018 22019881 PMC3254728

[32] Power JD, Mitra A, Laumann TO, Snyder AZ, Schlaggar BL, et al (2014) Methods to detect, characterize, and remove motion artifact in resting state fMRI. NeuroImage 84: 320–341. 10.1016/j.neuroimage.2013.08.048 23994314 PMC3849338

[33] Smyser CD, Inder TE, Shimony JS, Hill JE, Degnan AJ, et al (2010) Longitudinal analysis of neural network development in preterm infants. Cereb Cortex 20: 2852–2862. 10.1093/cercor/bhq035 20237243 PMC2978240

[34] Fair DA, Nigg JT, Iyer S, Bathula D, Mills KL, et al (2013) Distinct neural signatures detected for ADHD subtypes after controlling for micro-movements in resting state functional connectivity MRI data. Front Syst Neurosci 6: 80 10.3389/fnsys.2012.00080 23382713 PMC3563110

[35] Greicius MD, Krasnow B, Reiss AL, Menon V (2003) Functional connectivity in the resting brain: a network analysis of the default mode hypothesis. Proceedings of the National Academy of Sciences of the United States of America 100: 253–258. 10.1073/pnas.0135058100 12506194 PMC140943

[36] Stern ER, Fitzgerald KD, Welsh RC, Abelson JL, Taylor SF (2012) Resting-State Functional Connectivity between Fronto-Parietal and Default Mode Networks in Obsessive-Compulsive Disorder. PLoS ONE 7: e36356 10.1371/journal.pone.0036356 22570705 PMC3343054

[37] Taylor KS, Seminowicz DA, Davis KD (2009) Two systems of resting state connectivity between the insula and cingulate cortex. Hum Brain Mapp 30: 2731–2745. 10.1002/hbm.20705 19072897 PMC6871122

[38] Astafiev SV, Shulman GL, Stanley CM, Snyder AZ, Van Essen DC, et al (2003) Functional organization of human intraparietal and frontal cortex for attending, looking, and pointing. J Neurosci 23: 4689–4699. 12805308 10.1523/JNEUROSCI.23-11-04689.2003 PMC6740811

[39] Brier MR, Thomas JB, Snyder AZ, Benzinger TL, Zhang D, et al (2012) Loss of intranetwork and internetwork resting state functional connections with Alzheimer's disease progression. J Neurosci 32: 8890–8899. 10.1523/JNEUROSCI.5698-11.2012 22745490 PMC3458508

[40] Forman SD, Cohen JD, Fitzgerald M, Eddy WF, Mintun MA, et al (1995) Improved assessment of significant activation in functional magnetic resonance imaging (fMRI): Use of a cluster-size threshold. Magnetic Resonance in Medicine 33: 636–647. 7596267 10.1002/mrm.1910330508

[41] Binney RJ, Embleton KV, Jefferies E, Parker GJM, Ralph MAL (2010) The ventral and inferolateral aspects of the anterior temporal lobe are crucial in semantic memory: evidence from a novel direct comparison of distortion-corrected fMRI, rTMS, and semantic dementia. Cereb Cortex 20: 2728–2738. 10.1093/cercor/bhq019 20190005

[42] Miao X, Gu H, Yan L, Lu H, Wang DJJ, et al (2014) Detecting resting-state brain activity by spontaneous cerebral blood volume fluctuations using whole brain vascular space occupancy imaging. NeuroImage 84: 575–584. 10.1016/j.neuroimage.2013.09.019 24055705

[43] Aguirre GK, Zarahn E, D'Esposito M (1998) The variability of human, BOLD hemodynamic responses. NeuroImage 8: 360–369. 10.1006/nimg.1998.0369 9811554

[44] Birn RM, Saad ZS, Bandettini PA (2001) Spatial heterogeneity of the nonlinear dynamics in the FMRI BOLD response. NeuroImage 14: 817–826. 10.1006/nimg.2001.0873 11554800

[45] Handwerker DA, Roopchansingh V, Gonzalez-Castillo J, Bandettini PA (2012) Periodic changes in fMRI connectivity. NeuroImage 63: 1712–1719. 10.1016/j.neuroimage.2012.06.078 22796990 PMC4180175

[46] Obata T, Liu TT, Miller KL, Luh WM, Wong EC, et al (2004) Discrepancies between BOLD and flow dynamics in primary and supplementary motor areas: application of the balloon model to the interpretation of BOLD transients. NeuroImage 21: 144–153. 14741651 10.1016/j.neuroimage.2003.08.040

[47] Handwerker DA, Gonzalez-Castillo J, D'Esposito M, Bandettini PA (2012) The continuing challenge of understanding and modeling hemodynamic variation in fMRI. NeuroImage 62: 1017–1023. 10.1016/j.neuroimage.2012.02.015 22366081 PMC4180210

[48] Bandettini PA, Wong EC (1995) Effects of biophysical and physiologic parameters on brain activation-induced R2 * and R2 changes: simulations using a deterministic diffusion model. International Journal of Imaging Systems and Technology 6: 133–152.

[49] Boxerman JL, Hamberg LM, Rosen BR, Weisskoff RM (1995) Mr contrast due to intravascular magnetic susceptibility perturbations. Magnetic Resonance in Medicine 34: 555–566. 10.1002/mrm.1910340412 8524024

[50] Weisskoff RM, Zuo CS, Boxerman JL, Rosen BR (1994) Microscopic susceptibility variation and transverse relaxation: theory and experiment. Magn Reson Med 31: 601–610. 8057812 10.1002/mrm.1910310605

[51] Aguirre GK, Detre JA, Zarahn E, Alsop DC (2002) Experimental design and the relative sensitivity of BOLD and perfusion fMRI. NeuroImage 15: 488–500. 10.1006/nimg.2001.0990 11848692

[52] Tak S, Wang DJJ, Polimeni JR, Yan L, Chen JJ (2014) Dynamic and static contributions of the cerebrovasculature to the resting-state BOLD signal. NeuroImage: 1–9. 10.1016/j.neuroimage.2013.09.057 PMC4323159 24099842

[53] Van Dijk KRA, Hedden T, Venkataraman A, Evans KC, Lazar SW, et al (2010) Intrinsic Functional Connectivity As a Tool For Human Connectomics: Theory, Properties, and Optimization. Journal of Neurophysiology 103: 297–321. 10.1152/jn.00783.2009 19889849 PMC2807224

[54] Franco AR, Mannell MV, Calhoun VD, Mayer AR (2013) Impact of Analysis Methods on the Reproducibility and Reliability of Resting-State Networks. Brain Connectivity 3: 363–374. 10.1089/brain.2012.0134 23705789 PMC3749744

[55] Andrews-Hanna JR, Snyder AZ, Vincent JL, Lustig C, Head D, et al (2007) Disruption of Large-Scale Brain Systems in Advanced Aging. Neuron 56: 924–935. 10.1016/j.neuron.2007.10.038 18054866 PMC2709284

[56] Honey CJ, Sporns O, Cammoun L, Gigandet X, Thiran JP, et al (2009) Predicting human resting-state functional connectivity from structural connectivity. Proceedings of the National Academy of Sciences of the United States of America 106: 2035–2040. 10.1073/pnas.0811168106 19188601 PMC2634800

[57] Fornito A, Zalesky A, Breakspear M (2013) Graph analysis of the human connectome: Promise, progress, and pitfalls. NeuroImage 80: 426–444. 10.1016/j.neuroimage.2013.04.087 23643999

[58] Bandettini PA, Wong EC, Jesmanowicz A, Hinks RS, Hyde JS (1993) Simultaneous mapping of activation-induced ΔR2* and ΔR2 in the human brain using a combined gradient-echo and spin-echo EPI pulse sequence. Proceedings of the 12th Annual Meeting of the Society of Magnetic Resonance in Medicine: 169.

[59] Pruessmann KP, Weiger M (1999) SENSE: sensitivity encoding for fast MRI. Magnetic Resonance in Medicine 42: 952–962. 10542355

